# Mutation-based, short-term “neoadjuvant” treatment allows resectability in stage IVB and C anaplastic thyroid cancer

**DOI:** 10.1007/s00405-023-07827-y

**Published:** 2023-01-13

**Authors:** Elisabeth Maurer, F. Eilsberger, S. Wächter, J. Riera Knorrenschild, A. Pehl, K. Holzer, A. Neubauer, M. Luster, D. K. Bartsch

**Affiliations:** 1 Department of Visceral-, Thoracic- and Vascular Surgery, Philipps-University Marburg, Baldingerstraße, 35043 Marburg, Germany; 2 Department of Nuclear Medicine, Philipps-University, Marburg, Germany; 3 Department of Internal Medicine, Hematology, Oncology and Immunology, Philipps-University, Marburg, Germany; 4 Institute of Pathology, Philipps-University, Marburg, Germany

**Keywords:** Anaplastic thyroid cancer, Neoadjuvant therapy, Mutation-based therapy

## Abstract

**Introduction:**

Few available data indicate that a mutation-based “neoadjuvant” therapy in advanced anaplastic thyroid carcinoma (ATC) might convert an initially unresectable primary tumor to resectable and optimize local tumor control. We evaluated a preoperative short-term “neoadjuvant” therapy with a BRAF-directed therapy or, in case of BRAF non-mutated tumors, an mKI/checkpoint inhibitor combination in three patients with ATC stage IVB and C.

**Methods:**

In the context of preoperative diagnostics, immunohistochemistry (IHC) assessment and genetic analysis was started as soon as possible. The antiangiogenetic therapy with lenvatinib was immediately after diagnosis of ATC started as bridging therapy. In case of a BRAF-mutated ATC, a combination therapy of dabrafenib and trametinib, in case of BRAF-wildtype ATC a combination of pembrolizumab and lenvatinib was given for 4 weeks. If re-staging has shown a significant therapy response due to a decrease in size of > 50%, surgical resection was reconsidered. A primary tumor resection was performed first. As a second step, limited distant metastasis have been resected approximately 4 weeks after thyroid surgery. After postoperative recovery, the targeted systemic therapy was continued.

**Patients:**

Two patients presented with BRAF-wildtype ATC stage IVC, one with BRAF-mutated ATC stage IVB. All patients were evaluated by surgery, nuclear medicine and oncology upon diagnosis of ATC.

**Results:**

In all three cases, the “neoadjuvant” therapy induced a dramatic response and led to local resectability in primarily non-resectable ATC stage IVB or C. We have chosen for the first time a short-term “neoadjuvant” treatment period to reduce the risk of bleeding and/or fistula due to potential rapid tumor shrinkage. The results of surgery after only short-term “neoadjuvant” therapy showed two R0 und one R1 resections. Postoperative histopathological findings confirmed an extent of tumor necrosis or regressive fibrotic tissue between 60 and > 95% in our patients.

**Conclusions:**

A short-term mutation-based “neoadjuvant” therapy can achieve local resectability in initially unresectable ATC stage IVB or C. A neoadjuvant treatment period of about 4 weeks seems to show similar response as a treatment duration of at least 3 months.

## Introduction

Anaplastic thyroid carcinoma (ATC) is one of the rarest thyroid malignancies with an incidence of 1–2 patients per million people per year, but it is a highly aggressive entity with a mortality of up to 100% [1–3]. Available retrospective data confirm a poor prognosis of ATC with a median overall survival (OS) after initial diagnosis of 3–6 months and a median 1-year survival of about 20% of patients [1, 4, 5].

Mutation-guided individualized therapeutic strategies have been applied in advanced or initially unresectable ATC [3]. Retrospective assessments of outcomes observed from using this strategy have recently been published, with emerging evidence to support a potential survival advantage to the use of targeted therapy in ATC [6].

In ATC stage IVA and resectable stage IVB upfront surgery with the goal of a R0 or R1 resection is the preferred therapy often followed by external beam radiation and chemotherapy or a targeted therapy according to tumor genetics within clinical trials [3]. In initially unresectable stage IVB and metastatic stage IVC early initiation of cytotoxic chemotherapy with anthracyclines, taxanes and platinum is suggested as an initial and potentially “bridging approach” until mutational data and/or mutationally specified therapies might be available [3, 6]. In case of tumor response, secondary surgery can be considered if feasible.

The idea of neoadjuvant treatment with chemotherapy for ATC IVB and C followed by a tumor resection in the course was considered by several study groups in the past [7–11]. The option to use a targeted, mutation-based therapy in a “neoadjuvant” setting in ATC stage IVB or C might convert an unresectable primary tumor to resectable. Maniakas et al. recently reported their nearly 20-year experience in treatment of 479 ATC patients. 23 of 479 patients received surgery after a neoadjuvant treatment, 20 of them BRAF-directed therapies, the other 3 patients underwent chemotherapy or a checkpoint-/MEK-inhibitor combination. In this retrospective analysis, the overall survival of neoadjuvant treated patients with BRAF-directed therapies followed by surgery (*n* = 20) was significantly prolonged compared to the patients treated with a BRAF-/MEK-inhibitor combination without tumor resection (*n* = 35) (1-year survival 94% vs. 52%, *p* = 0.02) Eight of these 20 patients presented with stage IVC disease [6]. A case report of Cabanillas et al. described a neoadjuvant treatment with the BRAF-/MEK-inhibitor combination dabrafenib plus trametinib after a bridging therapy with paclitaxel and carboplatin in a stage IVB ATC. The neoadjuvant therapy was extended after tumor progression to pembrolizumab, a checkpoint inhibitor, due to a high programmed death ligand 1 (PD-L1) expression of the respective tumor. The neoadjuvant therapy was followed by thyroidectomy and neck dissection and resulted in a R1 resection [12]. A case series of 6 patients stage IVB (*n* = 4) and IVC (*n* = 2) showed a R0 resection in 4 and a R1 resection in 2 patients after neoadjuvant therapy with dabrafenib and trametinib. Two patients were additionally treated with pembrolizumab preoperatively. After a median follow-up of 15 months from start of the BRAF-directed therapy four patients are alive without evidence of disease (NED) and two patients died of disease (DOD) [13]. McCrary et al. reported a targeted neoadjuvant therapy in four patients. Two patients with a BRAF^V600E^ mutation were treated with a dual BRAF/MEK-inhibition, two patients with BRAF-wildtype ATC received the checkpoint inhibitor pembrolizumab in combination with the multikinase inhibitor (mKI) lenvatinib. There was a clinical and radiological response, one patient underwent thyroidectomy and neck dissection (ypT1 ypN0) [14].

Thus, it has been suggested that a preoperative “neoadjuvant” therapy with a BRAF-directed therapy or, in case of BRAF non-mutated tumors, a mKI/checkpoint inhibitor combination can lead to tumor resectability in locally advanced ATC and optimize local tumor control. Here, we report the results of a successful mutation-based off-label “neoadjuvant” treatment in three patients with ATC stage IVB and C.

## Methods

In case of clinical suspicion for ATC the first step at our institution was a core needle biopsy [15] as the cytological diagnosis with fine needle aspiration of ATC can be challenging [16]. Immunohistochemistry (IHC) assessment was started as soon as possible. Thyroid-specific proteins such as thyroglobulin (Tg) and thyroid-lineage markers such as thyroid-transcription factor 1 (TTF-1) were determined. In general, they are absent in ATC, reflecting the undifferentiated nature of the tumor [17]. PAX-8 expression was identified [17]. Cellular proliferation was assessed by Ki-67 [17]. Nuclear staining of p53 was measured. Loss of p53 tumor suppressor function via somatic mutation of TP53 was determined as typical in ATC and as differentiation from poorly differentiated thyroid carcinoma (PDTC) [18]. BRAF mutation status was determined as well as mutation in RAS-family genes by molecular analysis [17–20]. PD-L1 expression was measured to clarify the possibility of a pembrolizumab therapy as drugs targeting the interaction between the programmed death-1 (PD-1) receptor and its ligand, programmed death ligand 1and 2 (PD-L1/L2), have shown clinical activity in ATC with high PD-L1 expression [21–23].

Parallel we started imaging procedures. Radiological tumor staging was done by MRI of the neck to assess a local infiltration and ^18^F-FDG–PET/CT of the whole body for evaluation of metastasis. Esophagoscopy and tracheoscopy were preformed to clarify intraluminal tumor growth. A preoperative laryngoscopy was performed to assess a potential vocal cord palsy.

Once the result of immunohistochemistry and molecular testing were available, all patients with ATC were discussed in the multidisciplinary tumor conference with focus on patient clinical condition and comorbidities. The off-label use of a targeted “neoadjuvant” therapy proposed by the board was initiated immediately. Patients were informed in detail about the therapy and informed consent was obtained from all three patients. In case of a BRAF-mutated ATC, a combination therapy of dabrafenib and trametinib, in case of BRAF-wildtype ATC a combination of pembrolizumab and lenvatinib was given. The mKI lenvatinib is since 2015 approved in Europe (EMA European Medicines Agency) for several tumor entities among them the therapy of differentiated iodine refractory thyroid carcinoma. The checkpoint inhibitor therapy with pembrolizumab and the BRAF/MEK inhibitor therapy with dabrafenib and trametinib has not been approved in Europe for the therapy of ATC. An individual approval for the respective therapy was requested from the distinctive health insurance, which was approved in all cases. Therefore, we started the antiangiogenetic therapy with lenvatinib 20 mg daily immediately as bridging and combined it with pembrolizumab (200 mg q3 weeks) or switched to trametinib 4 mg daily/dabrafenib 150 mg twice a day after a successful approval. The individual, mutation-based therapy was continued for 4 weeks.

The proposed concept in each patient was as follows. After a limited period of 4-week re-staging was done using ^18^F-FDG–PET/CT. In case of significant therapy response due to a decrease in size of > 50%, surgical resection was reconsidered. This short period of medical treatment time was chosen to reduce the risk of bleeding and/or fistula due to potential rapid tumor shrinkage based on the therapy with lenvatinib [24–26].

If there was a regression in size or glucose metabolism, a primary tumor resection was performed first. As a second step, limited distant metastasis should be resected approximately 4 weeks after thyroid surgery. After postoperative recovery of 2 weeks, the targeted systemic therapy should be continued.

## Results

Between November 2021 and May 2022 three patients with stage IVB (one patient) and IVC (two patients) underwent mutation-based, short-term “neoadjuvant” treatment, followed by tumor resection.

### Patient 1#

**Fig. 1:**
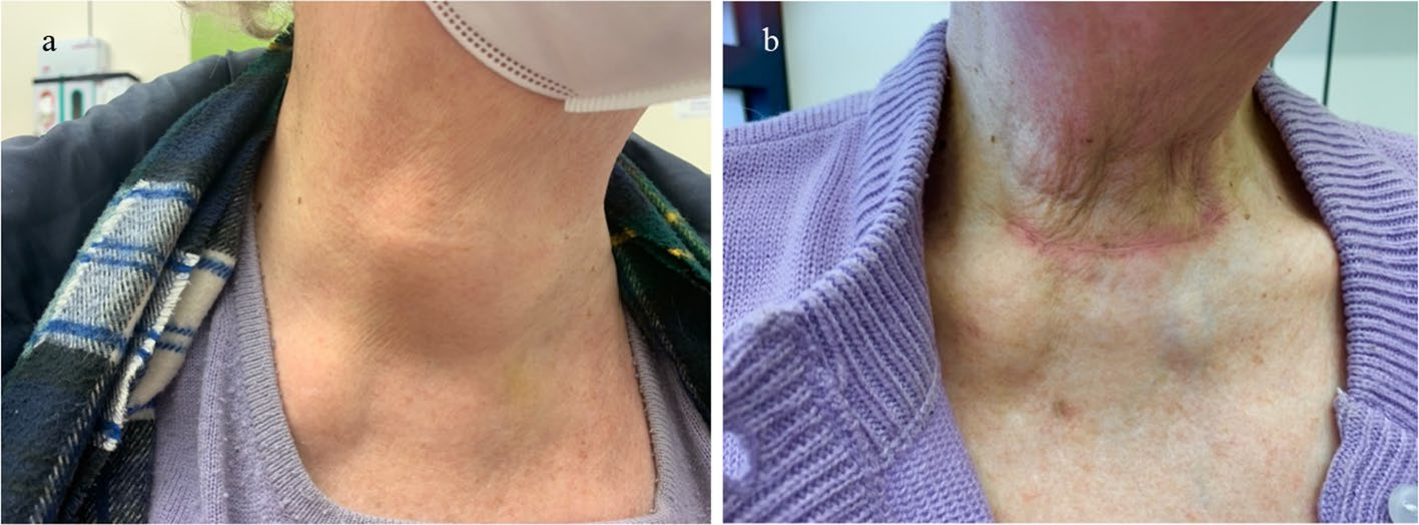
Patient 1# with ATC of the left thyroid lobe and isthmus: local findings pre-neoadjuvant treatment (**a**) and postoperatively (**b**)

An 81-year-old female presented with a fast-growing thyroid mass on the left side and isthmus and hoarseness (Fig. 1). Core needle biopsy resulted in a BRAF-wildtype ATC, with an expression of PD-L1 of 80% (Table 1). MRI demonstrated a left-sided thyroid tumor with likely tracheal invasion and esophageal compression and thrombosis of the left internal jugular vein. Local central and lateral left sided lymph node metastases were also visualized. ^18^F-FDG–PET/CT showed a hypermetabolic lesion affecting the left thyroid lobe and isthmus, a left-sided lymphadenopathy and one pulmonary metastasis in the right middle lobe. Bronchoscopy confirmed a tumor infiltration of the left subglottic trachea, esophagoscopy was without evidence of tumor infiltration findings. A preoperative laryngoscopy confirmed a paralysis of the left vocal cord.

**Table 1:** Immunohistochemistry and mutation analysis of patients with ATC

	Patient 1#	Patient 2#	Patient 3#
Immunohistochemistry			
Tg IMH	Neg	Neg	Neg
TTF1 IMH	Neg	Neg	Neg
Ki-67 IMH	80%	40%	75%
p53 IMH	Overexpression	Overexpression	Overexpression
Pax8 IMH	Neg	Pos	Pos
PD-L1 IMH	TPS score 80% CPS score 80	TPS score 90% CPS score 92	TPS score 80% CPS score 90
Genetic mutations			
BRAF	Neg	Neg	p.Val600Glu
NRAS	Neg	p.Gln61Arg	neg
TP53	C277	p.Pro278Ser	n.a
Fusion ALK	Neg	Neg	Neg
ROS1	Neg	Neg	Neg
NTRK1-3	Neg	Neg	Neg
MET exon 14 skipping	Neg	Neg	Neg

*IMH* immunohistochemistry, *TPS* tumor proportion score, *CPS* combined positivity score

**Fig. 2:**
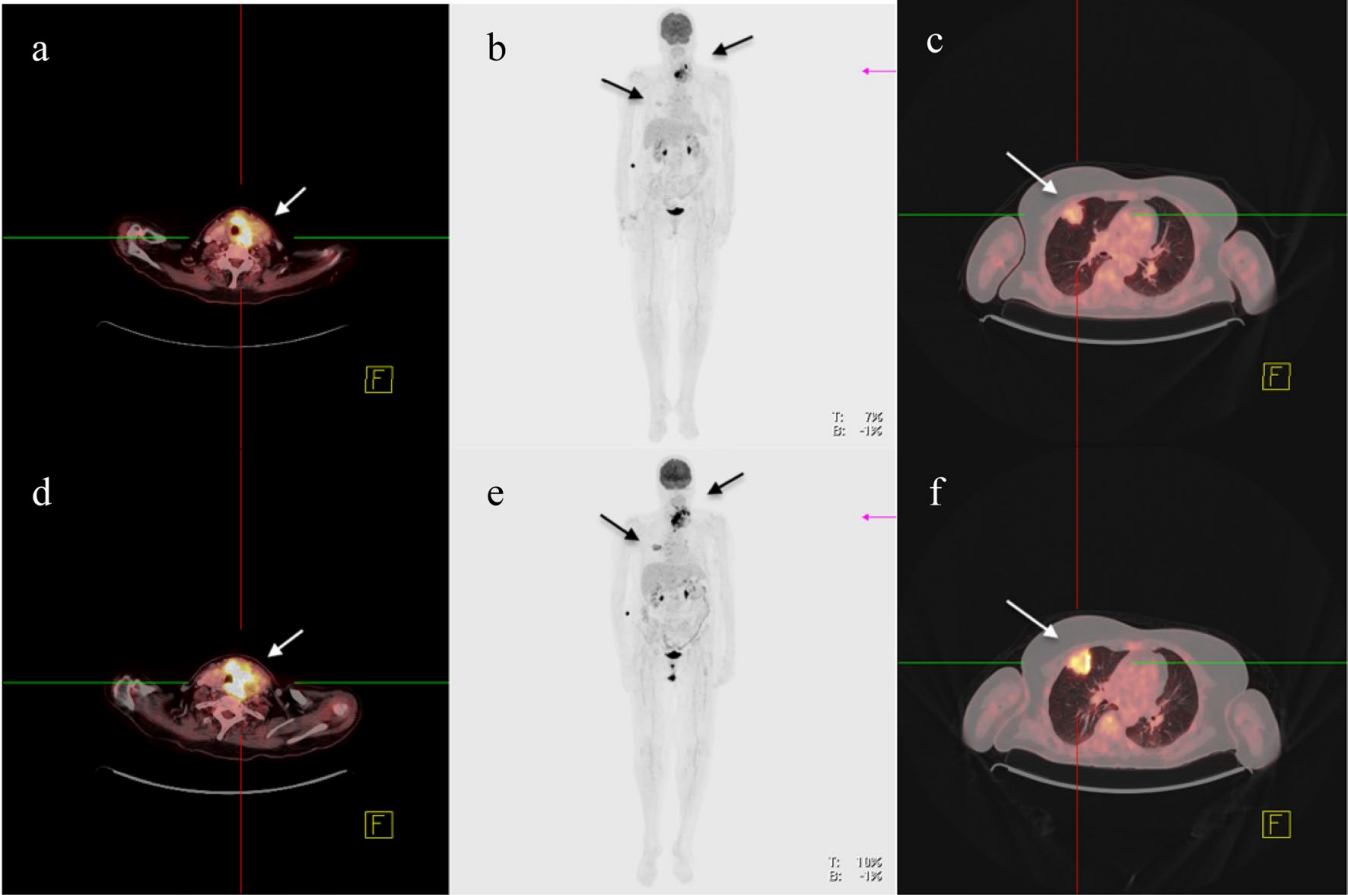
Patient 1# ATC of left thyroid lobe and isthmus and singular pulmonary metastasis of the middle lobe: ^18^F-FDG–PET/CT (**d**–**f**) at diagnosis and after neoadjuvant therapy (**a**–**c**). **a**/**d** Hypermetabolic mass affecting the left thyroid lobe and isthmus after neoadjuvant treatment/at diagnosis. **b**/**e** Primary tumor and pulmonary metastasis after neoadjuvant treatment/at diagnosis. **c**/**f** Pulmonary metastasis in the right middle lobe after neoadjuvant treatment/at diagnosis

We started a neoadjuvant therapy with Lenvatinib 20 mg daily for 4 weeks (Table 2). The mKI therapy was well-tolerated by the patient and there were no side effects. The ^18^F-FDG–PET/CT after neoadjuvant therapy had shown a dramatic regression of both, primary tumor and pulmonary metastasis, and a markedly decreased FDG-uptake (Fig. 2). The initial tumor infiltration was no longer evident in tracheoscopy. The approval for pembrolizumab by the patient’s health insurance company was not yet available after 4 weeks. Thus, after 26-day lenvatinib was paused and 7 days later total thyroidectomy was performed with central and left lateral lymph node dissection und resection of the thrombosed internal jugular vein on the left side. In addition, the patient underwent limited resection of the esophageal muscularis and we shaved the tumor from the trachea. The recurrent laryngeal nerve was walled by tumor und consecutively resected.

**Table 2:** Patients’ demographics and treatment data

Patient #	Age at dx	Gender	Medications utilized for neoadjuvant therapy	Length of neoadjuvant treatment	Surgical treatment	Final pathology	Postoperative complications/discharge	Follow-up (mo.)
1	81	F	lenvatinib 20 mg daily	lenvatinib 26 days	TTX + LAD, resection of RLN, the thrombosed internal jugular vein, limited resection of the esophageal muscularis, shave resection of the trachea Followed by thoracotomy, atypical resection of 1 pulm. Metastasis	ypT4a ypN1b (4/15) L0 V0 Pn1 R1(locoregional) ypM1 (pulm)	non POD 6	6, DOD
2	57	M	lenvatinib 20 mg daily, pembrolizumab 200 mg q3 weeks	lenvatinib 32 days pembrolizumab 2x	TTX + LAD, resection of the left internal jugular vein, shave resection of the trachea	ypT4a ypN 0(0/42) L0 V1 Pn0 R0	non POD 7	11, AWD
3	73	M	lenvatinib 20 mg daily, dabrafenib 150 mg twice a day trametinib 4 mg daily	lenvatinib 18 days dabrafenib/trametinib 25 days, of which reduced dose of dabrafenib 75 mg twice a day for 7 days	TTX + LAD right central compartment	ypT3b ypN0(0/9) L0 V1 Pn1 R0	non POD 5	8, AWD

*RLN* recurrent laryngeal nerve, *POD* postoperative day, *TTX* total thyroidectomy, *LAD* lymphadenectomy, *DOD* death of disease, *AWD* alive with disease

**Fig. 3:**
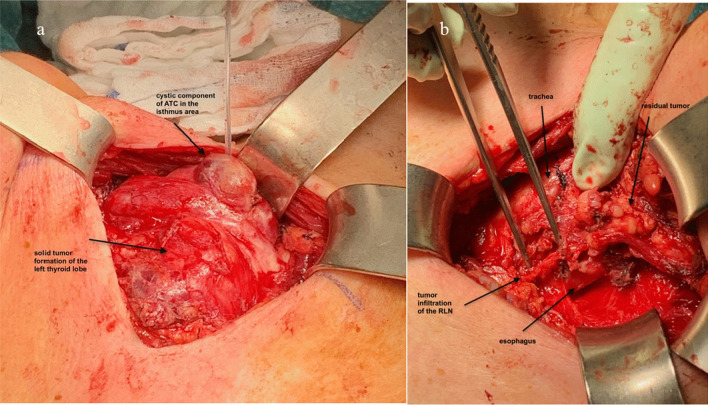
Patient 1# intraoperative situs in the course of thyroidectomy. **a** ATC of the left thyroid lobe and isthmus after Kocher incision. **b** Situs after subtotal tumor resection, infiltrated recurrent laryngeal nerve and tracheal wall

The histopathological examination showed an R1-resection (former subglottic tracheal infiltration), ypT4a ypN1b (4/15), L0 V0 Pn1 R1 (locoregional) ypM1 (pulmo) (Fig. 3). The postoperative course was uneventful, the wound healing was unimpaired (Fig. 2). The patient was discharged on postoperative day (POD) 6.

Two weeks after thyroidectomy we performed a thoracotomy on the right side and a wedge resection of the middle lobe for removal of the pulmonary metastasis. The patient did well-postoperatively without any complications. After complete wound healing the adjuvant therapy with lenvatinib was continued 14 days after the lung resection. In addition, the now approved pembrolizumab therapy was started. The follow-up ^18^F-FDG–PET/CT 4 months after thoracotomy showed a local glucose uptake in the right paratracheal area suspicious for a limited local recurrence. Pembrolizumab was not tolerated due to allergic dermatitis and skin detachment. A chemotherapy was not given because of the patient’s wishes and a reduced general condition. The patient received a radiation therapy. She died 6 months postoperatively and 8 months after diagnosis from local tumor complications (Table 2).

### Patient 2#

A 57-year-old male presented with hoarseness and weight loss of 8 kg within the last 2 months. After diagnosis of recurrent laryngeal nerve palsy on the left side, an ambulant computed tomography of the neck was initiated by a resident otolaryngologist and showed a left-sided thyroid tumor with suspicion of extrathyroidal extension. Core needle biopsy confirmed a BRAF-wildtype ATC. PD-L1 expression was high with a TPS-score of 90% (Table 1).

The ^18^F-FDG–PET/CT showed a hypermetabolic mass in the left thyroid lobe, a lymphadenopathy on the ipsilateral neck and about 15 small (< 10 mm), but bilateral pulmonary metastases. Bronchoscopy confirmed a tumor infiltration of the left subglottic trachea, esophagoscopy was without sign of tumor infiltration.

We started a neoadjuvant therapy with lenvatinib 20 mg daily and pembrolizumab 200 mg (day 1, day 22). The therapy was well-tolerated by the patient and there were no side effects. A preoperative laryngoscopy confirmed again the recurrent laryngeal nerve palsy on the left side. After 32 days, lenvatinib was paused. Within this period pembrolizumab 200 mg was applied two times (Table 2).

**Fig. 4:**
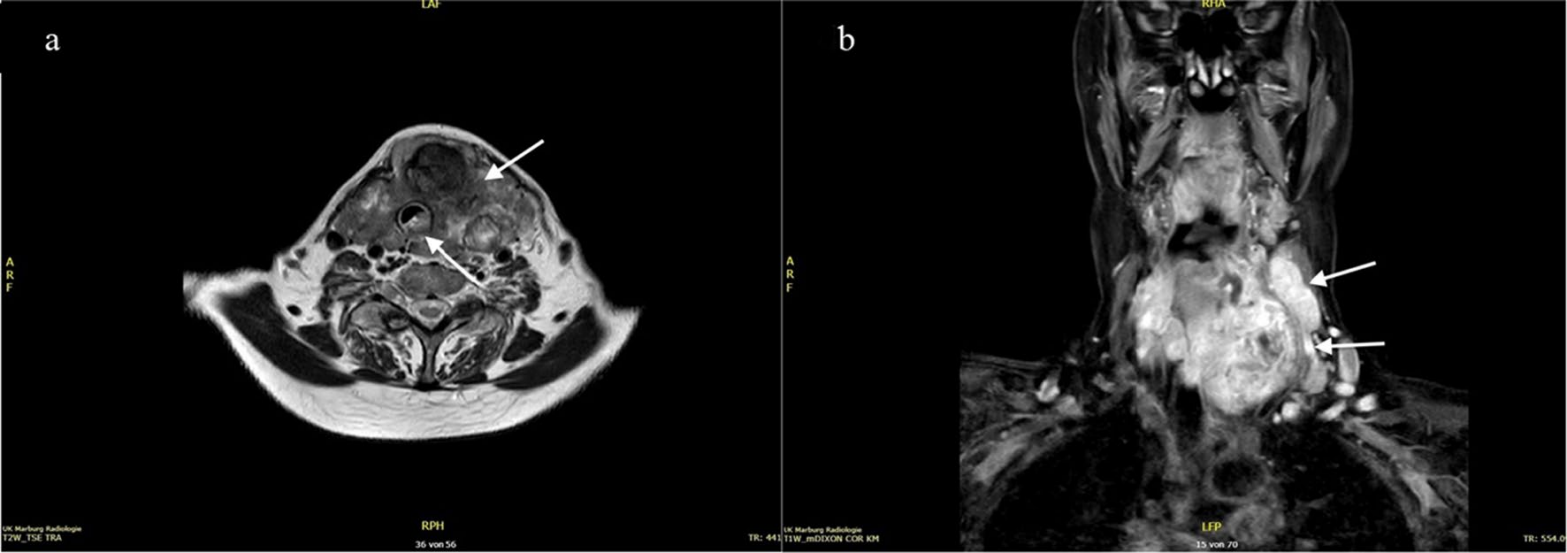
Patient 1# ATC of the left thyroid lobe: MRI at diagnosis. **a** Left-sided thyroid tumor with tracheal infiltration. **b** Carotid artery walled by tumor and lymph node metastases

Re-staging ^18^F-FDG–PET/CT showed a significant regression in size and FDG-uptake of the primary tumor and complete disappearance of the small bilobular lung metastases (Fig. 4). The pretherapeutic tumor infiltration of the trachea was no longer evident in a re-staging tracheoscopy.

Seven days after pausing the neoadjuvant therapy, we performed a total thyroidectomy with a shave resection of the trachea and lymphadenectomy on both sides of the central compartment and of the lateral compartment left. The left internal jugular vein was resected because of tumor thrombosis.

The histopathological examination showed extensive regressive necrosis in the area of the primary tumor with only few vital tumor cells. The edges of the tumor did not show any vital tumor cells, neither did lymph node metastases or the tumor thrombosis of the internal jugular vein. TNM classification was as follows: ypT4a ypN0(0/42) L0 V1 Pn0 R0 cTM1 (pulmo).

The postoperative course was uneventful, the wound healing was unimpaired. The patient was discharged on day 7 after surgery. Six days later, the lenvatinib therapy was continued. Another 4 days later pembrolizumab was given for the third time and continued every 3 weeks. Two months later the mKI/checkpoint inhibitor combination had to be stopped due to severe side effects (extensive colitis). Four months postoperatively, ^18^F-FDG–PET/CT raised suspicion of two small (1–1.5 cm) lymph node metastases in the right mediastinum. The adjuvant therapy was continued after recovery with carboplatin and paclitaxel. The patient is 11 months postoperatively alive with stable disease (Table 2).

### Patient 3#

A 73-year-old male presented for evaluation at our institution 2 weeks after undergoing an open biopsy in an outside hospital that revealed ATC. The surgery was discontinued because of irresectability. The initial symptoms were cervical pressure, hoarseness and hemoptysis. He reported a weight loss of 7 kg. A computed tomography was done in the outside hospital and showed an irregular lesion in the right thyroid lobe with compression of the trachea. Immunohistochemistry and mutation analysis was done on the specimen resected during the open biopsy. The tumor showed a BRAF^V600E^ mutation and high PD-L1 expression of 80%, Table 1). The ^18^F-FDG–PET/CT showed a large tumor of the right thyroid lobe and isthmus with suspicion of tracheal infiltration and an extensive lymphadenopathy on the ipsilateral neck. Distant metastases were not found. Tracheobronchoscopy with biopsy confirmed a tumor infiltration of the right subglottic trachea of about 1.5 cm with signs of intermittent bleeding. Esophagoscopy was without sign of tumor infiltration.

We started immediately before the results of next-generation sequencing were available a therapy with lenvatinib 20 mg daily under which the hemoptysis stopped within 5 days. When BRAF-mutation was confirmed and approval by the patient’s health insurance company, we switched the treatment to dabrafenib (150 mg twice a day) and trametinib (4 mg daily) after 4 weeks. In the course, the patient suffered of leukopenia and fever, so the therapy was paused for 3 days and afterwards dose of dabrafenib was reduced to 75 mg twice a day for 1 week. Then, the dose was increased up to the initial 150 mg twice a day and was well-tolerated. Dabrafenib and trametinib were given for 4 weeks (Table 2).

Re-staging with ^18^F-FDG–PET/CT only showed a residual local tumor without lymphadenopathy. Preoperative laryngoscopy confirmed an unimpaired vocal cord mobility, the initial tumor infiltration was no longer evident in tracheoscopy. Seven days after pausing the neoadjuvant therapy the patient underwent total thyroidectomy with shave resection of the trachea and lymphadenectomy of the central compartment on the right side.

**Fig. 5:**
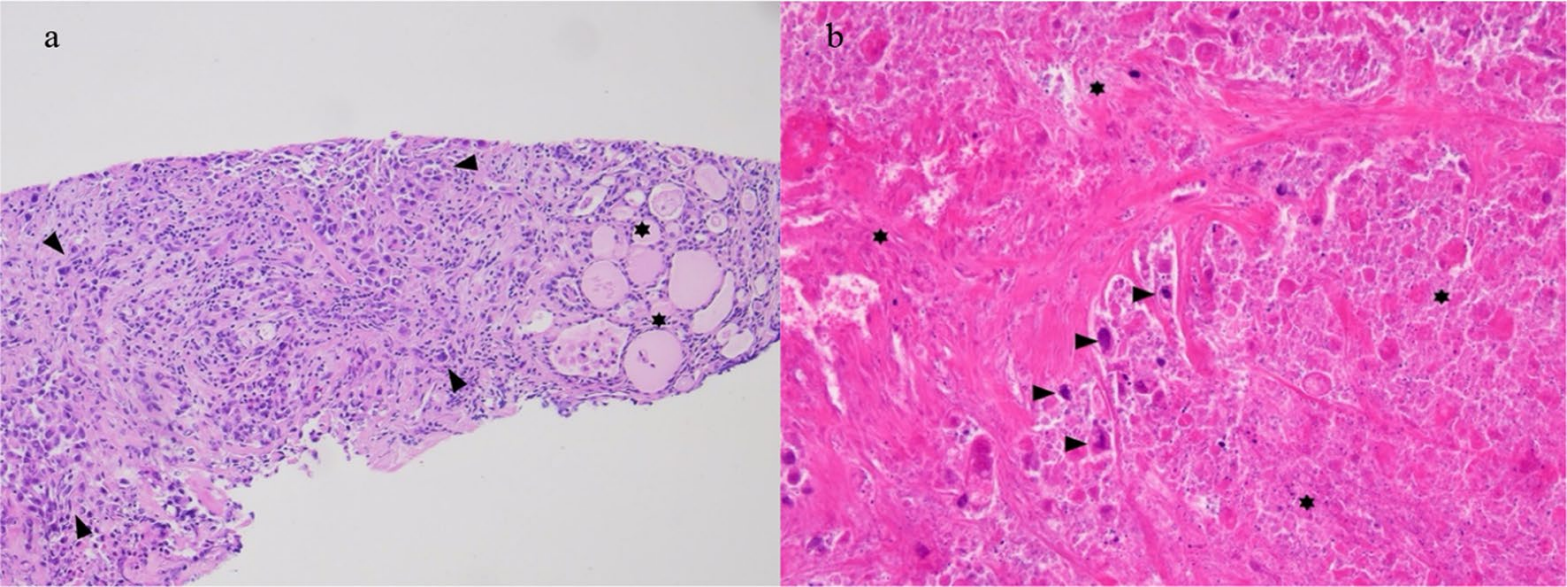
Representative images of tissue samples presenting histological regression (patient 2#). **a** Pre-therapeutic biopsy specimen of thyroid gland showing anaplastic carcinoma with undifferentiated pleomorphic tumor cells (marked by arrows) overgrowing residual thyroid parenchyma (marked by asterisks). Haematoxylin–eosin staining, magnification × 10. **b** Post-therapeutic resection specimen shows extensive areas of tissue necrosis in > 95% (marked by asterisks) with only sparse residual vital tumor cells (marked by arrows). Haematoxylin–eosin staining, magnification × 20

The histopathological examination showed extensive regressive necrosis in the primary tumor with only few vital tumor cells (Fig. 5). In the area of the former tracheal infiltration only inflammation and fibrosis were visible, but no more viable tumor cells. The TNM was as follows: ypT3b ypN0(0/9) L0 V1 Pn1 R0. The postoperative course was uneventful, the wound healing was unimpaired. The patient was discharged on day 5. The BRAF-directed therapy was continued 10 days after surgery. Six months postoperatively 18F-FDG–PET/CT showed a 1 × 1.5 cm local recurrence in the right thyroid bed. The systemic therapy was switched to lenvatinib and pembrolizumab. The patient is 8 months after surgery alive with stable disease.

## Discussion

In all three cases, the “neoadjuvant” therapy induced a dramatic response and led to local resectability in primarily non-resectable ATC stage IVB or C. When achievable, complete surgical resection has been shown to improve patients’ outcomes in ATC [27–30]. As most patients initially present with locally inoperable disease due to infiltration of carotid artery, larynx, trachea or esophagus, the possibility to achieve resectability with a “neoadjuvant” therapy is of great importance.

The option to use a mutation-based “neoadjuvant” treatment has shown response in several case series [6, 12–14]. Somatic alterations of BRAF are seen in ATC in 40–70% [18–20, 31]. 11–28% of ATCs express programmed death ligand-1 (PD-L1) in either the inflammatory background or the tumor cells themselves, which explains the potential response of ATC to immunotherapies [22, 23].

We have chosen for the first time a short-term “neoadjuvant” treatment period to reduce the risk of bleeding and/or fistula due to potential rapid tumor shrinkage as reported during the therapy with lenvatinib [24–26]. On the other hand, restaging with ^18^F-FDG–PET/CT after about 4 weeks of “neoadjuvant” treatment showed in all three patients a significant tumor reduction and reduced glucose uptake (Figs. 2, 4). This indicates that a short initial treatment period might be sufficient to achieve resectability. Wang et al. reported the course of six patients with BRAF-mutated, locally advanced ATC. The median duration of “neoadjuvant” treatment was 3.6 months (1.6–12). After that period, all patients received surgery and four times a R0 resection and two times a R1 resection could be achieved [13]. Our results of surgery after only short-term neoadjuvant therapy of 4–6 weeks were similar with two R0 und one R1 resections. McCrary et al. also described a “neoadjuvant” treatment in four patients, two with BRAF-mutated ATC and two with BRAF-wildtype ATC. So far, one patient received surgery after 4 months of therapy with dabrafenib and trametinib. The postoperative histopathological report stated stage ypT1ypN0. Two patients were at the time of publication still under “neoadjuvant” treatment (6 resp. 12 weeks) and one patient died of disease after 5 months.

Postoperative histopathological findings confirmed an extent of tumor necrosis or regressive fibrotic tissue between 60 and > 95% in our patients (Table 3). The case report from MD Anderson Cancer Center showed 30% necrosis after “neoadjuvant” treatment with dabrafenib monotherapy and in the course additionally pembrolizumab (three times) for 3 months [12]. This implies, a short neoadjuvant treatment period is sufficient for tumor regression.

**Table 3:** Histopathological pre-treatment findings vs. postoperative results

	Patient 1#	Patient 2#	Patient 3#
Pre-treatment histopathological findings	Highly cellular tumor tissue	Highly cellular tumor tissue with focal necrosis	Highly cellular tumor tissue with minimal sclerosed stroma
Postoperative histopathological findings	Extent tissue necrosis 60%, in between vital tumor cell nests	Extent of tissue necrosis < 95%, only few vital tumor cells	Regressive fibrotic tissue 60% with chronic inflammation, in between vital tumor cell nests

It remains unclear, if maintenance of BRAF/MEK inhibitors or immunotherapy in combination with mKI postoperatively is sufficient to avoid local recurrence or distant metastasis. An additional postoperative (chemo)radiation therapy might be considered but was not routinely administered. We decided to continue first of all with the treatment of BRAF/MEK inhibitors, respectively, mKI/checkpoint inhibitor combination. In our case series, all three patients developed a local recurrence or lymph node metastases. Thus, an adjuvant treatment with chemotherapy and radiation, possibly in combination with immunotherapy should be considered. An alternative treatment strategy would be personalized targeted therapies based on in vitro testing of patient-derived tumor tissue, for example, with tyrosine kinase inhibitors (TKIs) or the histone deacetylase inhibitor (HDACI) [32].

While awaiting access to off-label use of PD-L1 inhibitor or BRAF/MEK inhibitor, our patients were “only” treated with mKI. An additional bridging with chemotherapy (for example, carboplatin and paclitaxel) could possibly be effective to limit tumor growth.

In summary, a short-term mutation-based “neoadjuvant” therapy can achieve local resectability in initially unresectable ATC stage IVB or C. A neoadjuvant treatment of 4–6 weeks seems to show similar response as a treatment duration of at least 3 months and the short term might reduce the risk of life-threatening complications due to tumor shrinkage, such as trachea–esophageal fistula. The therapy of ATC remains challenging due to rapidly progressive tumor growths and the urgent need of an effective treatment, which additionally makes it difficult to enroll these patients in clinical trials. An individualized multimodal treatment will potentially prolong patients’ survival and optimize treatment options for future patients. The presented results of a mutation-based “neoadjuvant” treatment are highly promising and a prospective clinical trial to further analyze this new option is needed.

## Data Availability

The data that support the findings of this study are available from the corresponding author upon reasonable request.
